# Impact of KLF4 on Cell Proliferation and Epithelial Differentiation in the Context of Cystic Fibrosis

**DOI:** 10.3390/ijms21186717

**Published:** 2020-09-14

**Authors:** Luís Sousa, Ines Pankonien, Filipa B. Simões, Marc Chanson, Margarida D. Amaral

**Affiliations:** 1 BioISI—Biosystems & Integrative Sciences Institute, Faculty of Sciences, University of Lisboa, 1749-016 Lisbon, Portugal; lmdsousa@fc.ul.pt (L.S.); ipankonien@fc.ul.pt (I.P.); fbsimoes@fc.ul.pt (F.B.S.); 2 Departments of Pediatrics, Gynecology & Obstetrics and of Cell Physiology & Metabolism, Geneva University Hospitals and Medical School of the University of Geneva, 1211 Geneva, Switzerland; Marc.Chanson@unige.ch

**Keywords:** KLF4, wound healing, epithelial differentiation, proliferation, cystic fibrosis

## Abstract

Cystic fibrosis (CF) cells display a more cancer-like phenotype vs. non-CF cells. KLF4 overexpression has been described in CF and this transcriptional factor acts as a negative regulator of wt-CFTR. KLF4 is described as exerting its effects in a cell-context-dependent fashion, but it is generally considered a major regulator of proliferation, differentiation, and wound healing, all the processes that are also altered in CF. Therefore, it is relevant to characterize the differential role of KLF4 in these processes in CF vs. non-CF cells. To this end, we used wt- and F508del-CFTR CFBE cells and their respective KLF4 knockout (KO) counterparts to evaluate processes like cell proliferation, polarization, and wound healing, as well as to compare the expression of several epithelial differentiation markers. Our data indicate no major impact of KLF4 KO in proliferation and a differential impact of KLF4 KO in transepithelial electrical resistance (TEER) acquisition and wound healing in wt- vs. F508del-CFTR cells. In parallel, we also observed a differential impact on the levels of some differentiation markers and epithelial-mesencymal transition (EMT)-associated transcription factors. In conclusion, KLF4 impacts TEER acquisition, wound healing, and the expression of differentiation markers in a way that is partially dependent on the CFTR-status of the cell.

## 1. Introduction

Cystic fibrosis (CF) is the most common lethal genetic disease among Caucasians, with a variable geographic prevalence of 1:2500–6000 in Europe, according to the European Cystic Fibrosis Society registry [1]. CF is a multisystemic chronic and progressive disease caused by loss-of-function mutations in the CF transmembrane conductance regulator (*CFTR*) gene [2]. Over 2000 mutations in *CFTR* have been reported so far, but the deletion of the phenylalanine at position 508 (F508del) is by far the most common one, present in at least one allele in ~80% of individuals with CF worldwide. The F508del mutation impairs CFTR protein folding and plasma membrane (PM) trafficking, causing CFTR retention at the level of the endoplasmic reticulum, with only a minimal fraction reaching the PM with decreased function and stability [3]. CFTR has been shown to play a role in fundamental cellular processes related to differentiation, such as fetal development [4], epithelial differentiation/polarization [5], regeneration [6], and epithelial–mesenchymal transition (EMT) [7]. The multiple associations of CFTR and epithelial differentiation/EMT have been recently reviewed and reflect the idea that CF cells display a more cancer-like (vs. non-CF cells) phenotype due to the occurrence of a partial EMT [8], considered as a first stage into carcinogenesis [9]. Moreover, KLF4 has been linked to tumor metastasis through the regulation of EMT in several forms of human cancers [10].

The Kruppel-like factors (KLFs) comprise a family of evolutionarily conserved zinc finger transcription factors that regulate a variety of biological processes, including proliferation, differentiation, and apoptosis. In humans, 17 KLFs have been identified, of which KLF2, KLF4, and KLF5 have been linked to pluripotency [11]. Notably, KLF2, KLF4, and KLF5 have also been somewhat associated with CF [12,13,14,15,16,17,18,19]. Moreover, KLF4 has been described as overexpressed in F508del-CFTR CFBE cells, and it has been shown to act as a negative regulator of wt-CFTR (but not of F508del-CFTR) in a process mediated by AKT / GSK3β signaling [20]. KLF4 differential impact on CFTR levels and function may be due to the fact that KLF4 effects are often context-dependent [11].

KLF4 transcriptional profiling reveals its key role in cell-cycle regulation and epithelial differentiation [21]. Therefore, here we aim at understanding the role of KLF4 on cell proliferation, wound healing, EMT, and differentiation in the context of CF since these processes are disrupted in CF [6,8]. It has been demonstrated that KLF4 may exert very distinctive effects, depending on the cell context, i.e., its effects are dependent on the cell expression profile. For instance, KLF4 can function as an oncogene or a tumor suppressor depending on the type of cancer involved [22,23,24]. Indeed, KLF4 is often regarded as an inhibitor of cell proliferation [25] and as a tumor suppressor [26,27], as it is associated with both GSK3β [28] and AKT signaling pathways [29]. However, in certain contexts, KLF4 has also been shown to promote proliferation [30] and tumorigenesis [31,32], demonstrating its context-dependent roles.

Among its many effectors (see comprehensive list in [33]) is Epithelial-cadherin (E-Cad) [33]; we can expect a possible role of KLF4 in epithelial differentiation and wound healing, which is of potential interest in the CF context. For instance, KLF4 has been reported to transactivate promoters of epithelial genes like cytokeratin (CK) 19 [34]. Roles of KLF4 in differentiation have been reported in a variety of tissues. For example, KLF4 is required for lung differentiation [35] and epithelial barrier formation [36]. Moreover, KLF4 has been described as facilitating cutaneous wound healing by promoting fibrocyte generation [37]. Another study has shown that connexin (Cx) 26 overexpression due to KLF4 KO delayed epidermal barrier recovery [38]. Additionally, KLF4’s role in EMT has been extensively studied, being mostly associated with the negative regulation of EMT [39], but with some exceptions [24,39].

Therefore, our aim here is to characterize the role of KLF4 on proliferation, differentiation, and wound healing rate in the context of CF, using CF and non-CF KLF4 KO cell lines and their respective counterparts.

## 2. Results

### 2.1. KLF4 KO Impact on Proliferation

**Figure 1:**
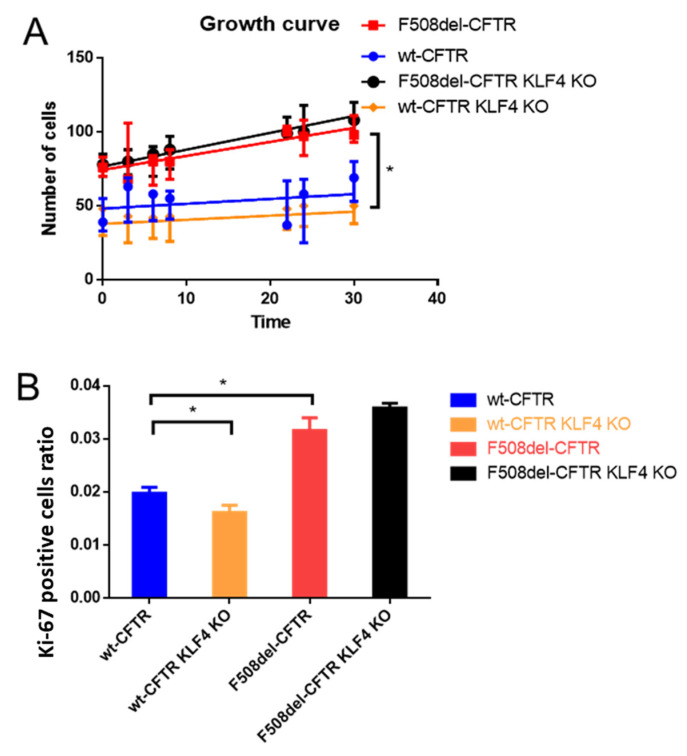
Impact of KLF4 knockout (KO) on cell proliferation, as assessed by growth curves and the proliferation marker Ki67 by immunofluorescence. (**A**) Growth curves of wt- and F508del-CFTR CFBE cells and their KLF4 KO counterparts (*n* = 3). (**B**) Ki-67 positive staining data, as assessed by the ratio of Ki67 positive cells over the total number of cells. (*n* = 3, unpaired *t*-test, asterisk is *p* < 0.05).

KLF4 KO has no major impact on cell proliferation, as shown by growth curves (Figure 1A), which reveal that proliferation is significantly higher in CF vs. non-CF cells (Figure 1A, red vs. blue line). Moreover, using the proliferative index biomarker Ki-67, we confirm the higher proliferation of CF cells (Figure 1B, red vs. blue) and also observe that the effect of KLF4 KO leads to significantly less proliferation of wt-CFTR cells (Figure 1B, yellow vs. blue bar). A trend, albeit nonsignificant, is observed for KLF4 KO in F508del-CFTR CFBE cells, leading to higher proliferation (Figure 1B, black vs. red bar), i.e., the opposite effect of what occurs in wt-CFTR cells. However, since the difference in F508del-CFTR CFBE cells is not statistically significant in both parameters (growth curve and Ki-67 staining), we conclude that KLF4 KO has no major impact on cell proliferation in the CF context. Accordingly, no major impact of KLF4 KO was observed in the expression of Ki-67, as assessed by Western blot (WB) (Figure S1).

### 2.2. KLF4 KO Impact on TEER and Wound Healing

**Figure 2:**
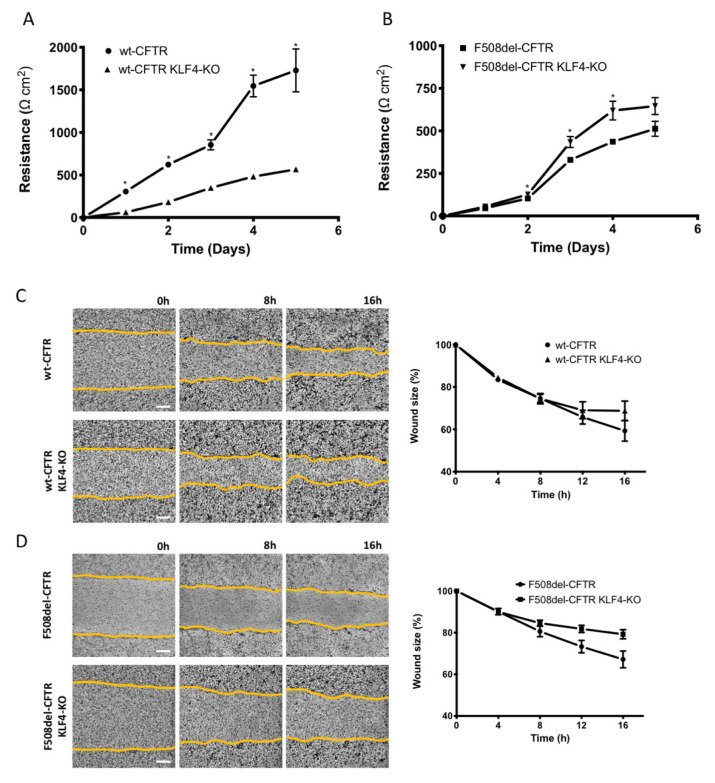
Impact of KLF4 KO on transepithelial electrical resistance (TEER) and wound healing. (**A**) TEER in wt- and (**B**) F508del-CFTR CFBE cells and their KLF4 KO counterparts over time. (*n* = 3, unpaired *t*-test, * is *p* < 0.05) (**C**) Representative images of wound healing over time (0, 8, and 16 h) on polarized wt-CFTR and wt-CFTR KLF4 KO and (**D**) polarized F508del-CFTR and F508del-CFTR KLF4 KO CFBE cells. Graphs (right panels) are showing the analysis of wound closure (wound size in %) over time (h). Data were normalized to the initial area of the wound and is represented as a percentage. Scale bar represents 300 μm. (*n* = 5).

We then assessed the impact of KLF4 KO in transepithelial electrical resistance (TEER) acquisition and wound healing. KLF4 KO significantly decreased TEER of wt-CFTR cells (indicating a leakier epithelium) whilst increasing TEER of F508del-CFTR cells (indicating a less leaky epithelium). Therefore, we observed, once again, a differential effect of KLF4 KO in the two cell lines (Figure 2A,B). Notably, we observed that wt-CFTR cells acquire higher TEER (vs. F508del-CFTR) (Figure 2A,B). We further performed wound healing experiments, and KLF4 KO also showed a differential impact on wt- vs. F508del-CFTR cells. While there is no major difference in the migration of the cells into the wound in wt-CFTR KLF4 KO vs. wt-CFTR cells, the KLF4 KO in F508del-CFTR cells shows a delay in wound closure (Figure 2C,D). We also stained the wound edges of the cells with actin in order to see if the forming of lamellipodia is affected by KLF4 (Figure S2). However, this does not seem to be the case, although we see differences in the actin cytoskeleton structure when comparing only wt- vs. F508del-CFTR cells.

### 2.3. KLF4 KO Impact on Differentiation Markers

**Figure 3:**
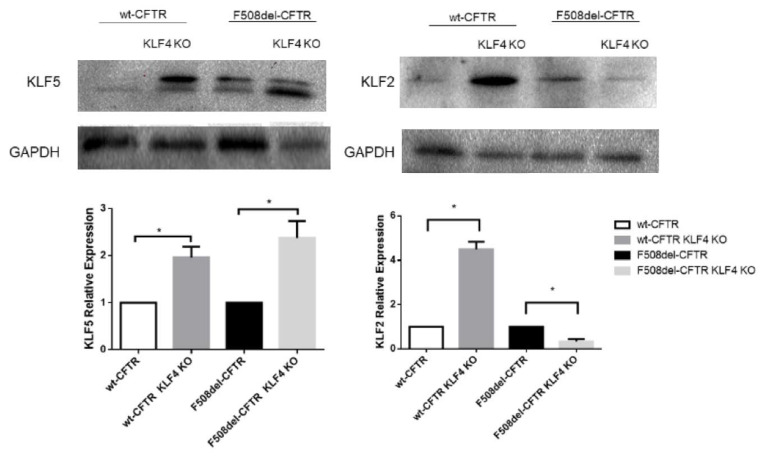
Impact of KLF4 KO on KLF2 and KLF5 levels. Protein levels in wt- and F508del-CFTR CFBE and their respective KLF4 KO counterparts. Representative WB of KLF2 and KLF5. GAPDH was used as loading control. Data are normalized to loading control and showed as relative expression (KO vs. non-KO; *n* = 3, unpaired *t*-test, * is *p* < 0.05).

We then investigated the impact of KLF4 KO on the levels of several epithelial differentiation markers and other KLF4-related transcription factors. Initially, we evaluated the effects of KLF4 KO on the expression levels of KLF2 and KLF5 (Figure 3). Our data show that KLF4 KO leads to KLF5 upregulation in both F508del- and wt-CFTR cells. Interestingly, the effects of KLF4 KO on KLF2 expression levels are different in CF vs. non-CF cells: KLF4 KO leads to increased KLF2 levels in wt-CFTR cells and decreased levels in F508del-CFTR cells.

**Figure 4:**
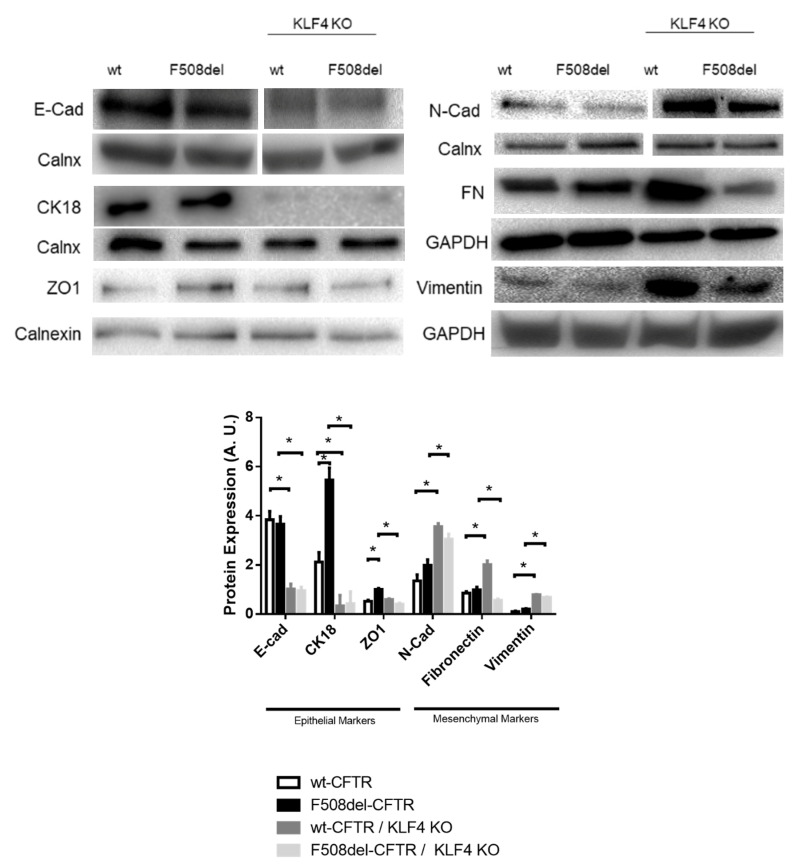
Impact of KLF4 KO on differentiation markers. Protein levels on wt- and F508del-CFTR CFBE and their respective KLF4 KO counterparts. Representative WB of E-Cad, N-Cad, CK18, ZO-1, fibronectin (FN), and vimentin. Calnexin, GAPDH, and vinculin were used as loading control. Data are normalized to loading control and showed as arbitrary units (A.U.; *n* = 3, unpaired *t*-test, * is *p* < 0.05).

KLF4 KO had a major impact on the levels of several differentiation markers (Figure 4). Indeed, KLF4 KO decreased the levels of E-Cad and cytokeratin 18 (CK18), which are commonly used as markers of epithelial cells, whilst increasing the levels of vimentin and Neural Cadherin (N-Cad), also commonly used as mesenchymal/EMT markers [40,41]. Therefore, KLF4 seems to be a suppressor of EMT. KLF4 KO, on the other hand, showed a differential impact on the expression levels of fibronectin (mesenchymal marker) and ZO-1 (zonula occludens-1, an epithelial marker) between wt- and F508del-CFTR CFBE cells. While KLF4 KO increased the expression levels of fibronectin in wt-CFTR cells, it led to a decrease in fibronectin in F508del-CFTR cells. Regarding ZO-1, KLF4 KO decreased its expression in F508del-CFTR CFBE cells, having no major impact on wt-CFTR CFBE cells. Notably, we observed that CF cells have increased levels of CK18 and ZO-1 (vs. non-CF cells).

**Figure 5:**
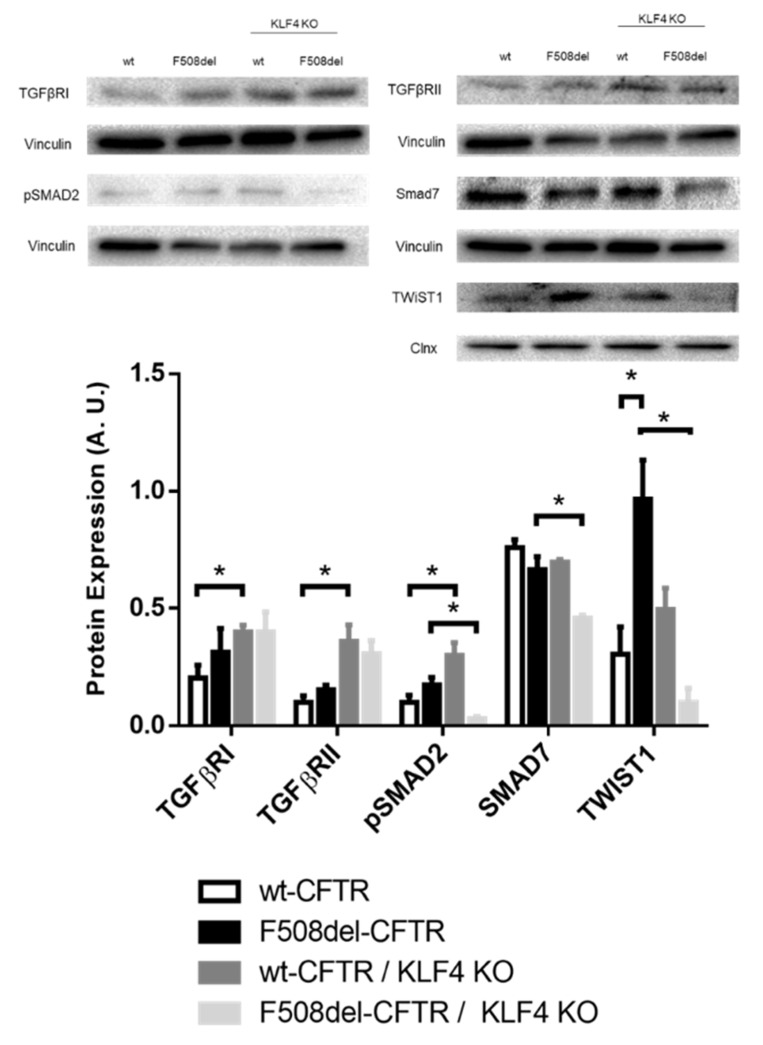
Impact of KLF4 KO on TGFβ pathway proteins and TWIST1. Protein levels on wt- and F508del-CFTR CFBE and their respective KLF4 KO counterparts. Representative WB of TGFβRI, TGFβRII, pSMAD2, SMAD7, and TWIST1. Calnexin and vinculin were used as loading control. Data are normalized to loading control and showed as arbitrary units (A.U.; *n* = 3, unpaired *t*-test, * is *p* < 0.05).

In terms of the impact of KLF4 KO on the levels of EMT-associated transcription factor (TF) TWIST1 (Figure 5), we observed that besides being upregulated in CF cells, KLF4 KO has a differential impact on its levels in wt- vs. F508del-CFTR CFBE cells. Indeed, while KLF4 KO had no major impact on the TWIST1 levels in wt-CFTR CFBE cells, it produced a marked decrease of this TF in F508del-CFTR CFBE cells. KLF4 KO also promotes the expression of TGFβ-related proteins (TGFβ receptors I and II), whose signaling is often associated with EMT in wt-CFTR CFBE cells (although a trend may also be observed in F508del-CFTR, albeit nonsignificant). Nevertheless, when we look at SMADs, phosphorylated (active) SMAD2 (pSMAD2) is differentially modulated by KLF4 KO (upregulated in wt- and downregulated in F508del-CFTR cells), and a significant decrease of SMAD7 was observed only in F508del-CFTR CFBE cells (Figure 5).

KLF4 KO showed no marked impact on the levels of other differentiation markers, such as Cx31, Occludin, and CK5, as well as other TFs such as SNAIL + SLUG (Figure S3).

Recently, immortalized human airway basal cells that retain multipotent differentiation capacity over long-term culture were developed (basal cell immortalized nonsmoking 1.1—BCi NS1.1). These cell lines display a tremendous potential for studying the processes underlying epithelial differentiation in the airways [42]. Having access to these cells, we decided to ascertain the expression of KLFs during BCi differentiation.

**Figure 6:**
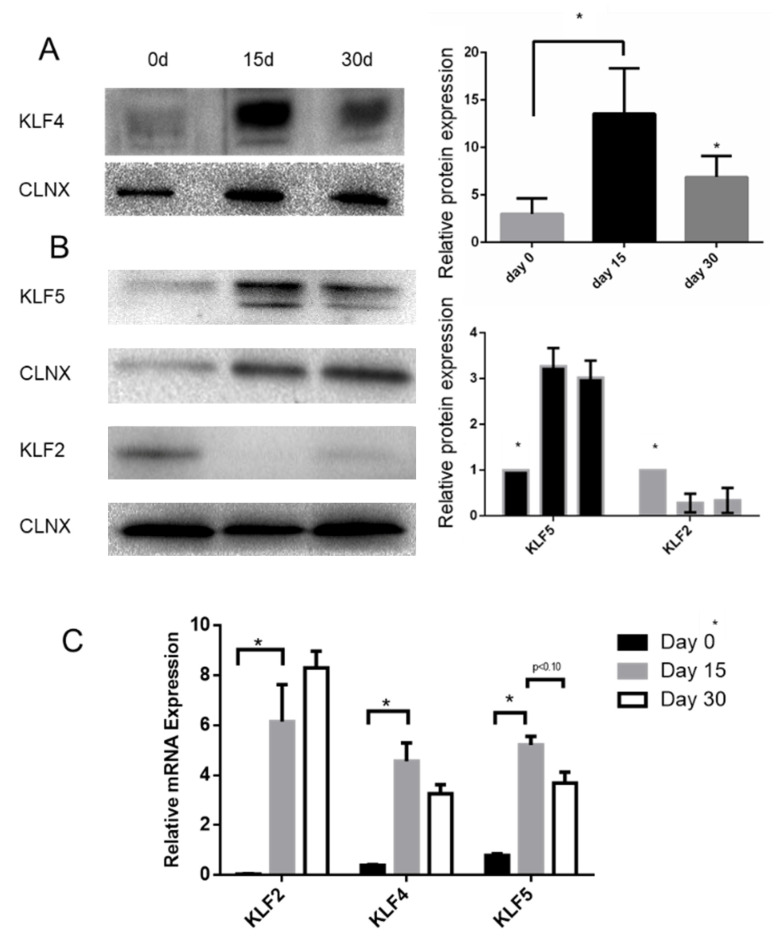
KLF2, KLF4, and KLF5 expression during BCi differentiation. KLF4 (**A**), KLF5, and KLF2 (**B**) expression during BCi differentiation. Representative KLF4, KLF2, and KLF5 Western blot (WB) images are shown. Calnexin was used as loading control. Data is normalized to loading control and showed as relative expression (vs. day 0) (**B**) or as arbitrary units (A.U.; *n* = 3, unpaired *t*-test, * is *p* < 0.05). (**C**) mRNA levels of *KLF2*, *KLF4*, and *KLF5* were assessed by qRT-PCR. Samples were retrieved during BCi differentiation (*n* = 3, unpaired *t*-test, * is *p*-value < 0.05).

Our data seem to indicate that KLF4 expression follows a pattern of expression that increases from day 0 to day 15 and has a slight decrease from then onwards (Figure 6A). We then decided to characterize the pattern of expression of KLF2 and KLF5 (Figure 6B). The data show that KLF5 expression follows a pattern of expression somewhat like the one observed for KFL4. However, KLF2 expression decreases with differentiation. qPCR data also confirm this pattern of expression for *KLF4* and *KLF5*, although having contrasting results in terms of the *KLF2* mRNA levels (Figure 6C).

**Figure 7:**
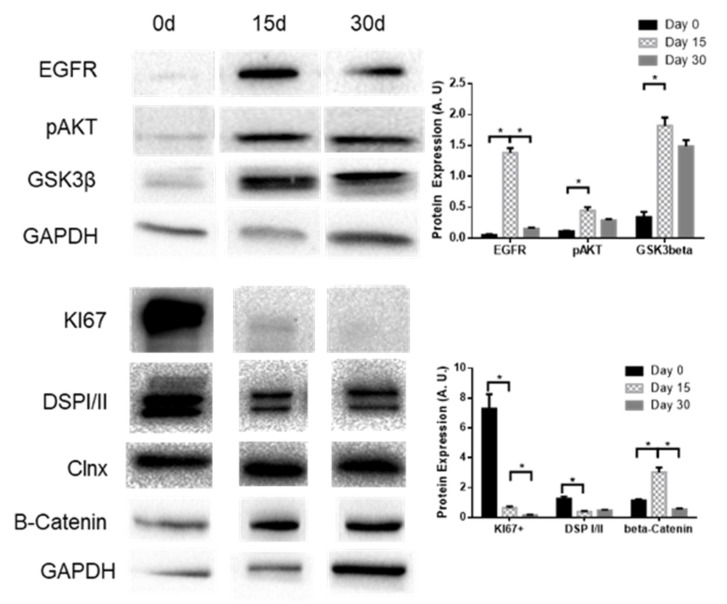
Expression levels of EGFR, pAKT, and GSK3β during BCi differentiation. Representative WB of EGFR, pAKT, GSK3β, Ki-67, DSPI/II, and β-catenin expression in BCi cells during differentiation. Calnexin and GAPDH were used as loading control. Data is normalized to loading control and showed as arbitrary units (A.U.; *n* = 3, unpaired *t*-test, * is *p* < 0.05).

Analysis of the levels of signaling pathways related to differentiation in BCi cells shows that β-catenin, EGFR, GSK3β, and pAKT levels mimic the KLF4 pattern of expression. Ki-67 and desmoplakin I/II (DSPI/II), on the other hand, seem to decrease their expression during differentiation (Figure 7).

## 3. Discussion

This work is aimed at characterizing the possible impact of KLF4 KO on proliferation, differentiation, and wound healing rate in the context of CF.

Our data (Figure 1 and Figure S1) show that KLF4 KO does not have a major impact on the proliferation rate of CFBE cells, as assessed by its impact on growth curves, Ki-67 positive staining, and Ki-67 levels assessed by WB. We expected, considering its role as a growth inhibitor [43], that KLF4 KO would induce a higher proliferation rate [25]. Nevertheless, our data show minimal changes in Ki-67 levels and growth rates, indicating that KLF4 by itself does not affect the proliferation rate in our system. We speculate that this may be due to cell-type specificity or due to the activation of compensatory mechanisms that keep the proliferation levels steady. In fact, we have seen that KLF4 KO induces overexpression of KLF5 (Figure 3), which may counteract the effects of KLF4 abrogation as KLF5 is often considered a promoter of proliferation [44].

We then evaluated the impact of KLF4 on epithelial integrity, as assessed by the acquisition of TEER, a measure of epithelial tightness, namely, at tight junctions and wound healing (Figure 2). Taken together, these data show, once again, that for a variety of cellular processes, KLF4 has different effects depending on the CFTR state of the cells. Indeed, KLF4 KO had opposing effects on TEER acquisition and wound healing rate in wt- vs. F508del-CFTR CFBE cells. In fact, in wt-CFTR CFBE cells, KLF4 KO promotes a “leakier” epithelium (by decreasing TEER), phenotypes often associated with a more mesenchymal state. Such an impact has been described previously [45]. Surprisingly, in F508del-CFTR cells, which are already more mesenchymal [7,8], KLF4 KO leads to the opposite effect, with cells acquiring higher levels of TEER (although not reaching wt-CFTR levels). We speculate that since CF cells start from a more mesenchymal state, this may influence the outcome of KLF4 KO. Noticeably, KLF4 KO impact on KLF2 expression is also dependent on the CFTR status (Figure 3), and KLF2 has been shown to mediate EMT [46]. Consistently, KLF4 has been shown to prevent EMT in human corneal epithelia [47] as well as in some types of lung cancers [48]. In fact, in some contexts, it has been shown that KLF4 actively promotes mesenchymal-to-epithelial transition, i.e., MET [49].

We also observed here that KLF4 KO decreases wound closure even further in F508del-CFTR cells, suggesting that KLF4 promotes wound healing. However, this is not the case in wt-CFTR, where KLF4-KO has no effect on wound closure. It has been shown in other cell lines (mouse fibroblasts) that KLF4 may have an impact on lamellipodia and actin organization [50], and we, therefore, stained the wound edges for actin (Figure S2). However, in our cell models, lamellipodia did not seem to be majorly affected by the knockout of KLF4.

Our data (Figure 3 and Figure 4) indicate that KLF4 KO has a profound impact on the levels of several differentiation markers and related transcription factors. Overall, these tend to indicate a shift towards a more mesenchymal phenotype in CF cells. Considering that F508del-CFTR cells are already more mesenchymal/cancer-like, we can then speculate that this transformation may have a higher relative impact on wt-CFTR cells. This may explain why KLF4 KO has a differential impact on the overall cellular processes in CF vs. non-CF cells. Since KLF4 has been shown to prevent EMT [47], the effect of its KO goes in accordance with what was expected, especially taking into account that KLF4 is a transcriptional factor of some of the evaluated markers, namely, E-cad, N-cad, and vimentin [39]. For instance, E-cad downregulation by decreasing levels of KLF4 has been previously reported [51] in parallel with the upregulation of vimentin and N-cad [39]. Additionally, KLF4 has been shown to promote CK18 expression [21]. Considering that KLF4 has a differential impact on CFTR levels and CFTR-dependent wound healing, mediators that are differentially regulated in wt- and F508del-CFTR may be of particular importance. Among the ones tested, ZO-1 and fibronectin are differentially modulated by KLF4 KO. In fact, ZO-1 regulation and signaling have been associated with CFTR, it having been demonstrated that ZO-1 directly interacts with CFTR to regulate epithelial differentiation [52]. Consistently, it has been reported that ZO-1 mislocalization and loss of function occur in the absence of CFTR, albeit with no impact on ZO-1 expression levels [53]. In fact, KLF4 KO has been previously shown to promote ZO-1 upregulation and mislocalization [54]. Regarding fibronectin, it has been previously reported to be upregulated in the secretome of CF bronchial epithelia [55], and its levels have been reported to be modulated by KLF4 [35]. Moreover, it may have an impact on CF pathology as it may affect *Pseudomonas aeruginosa* colonization [56]. Therefore, we can generally say that KLF4 KO induced a clear-cut EMT in wt-CFTR cells, but in F508del-CFTR cells, the KLF4 KO seems to promote EMT but has somewhat contradictory effects in certain mesenchymal markers/promoters, such as the upregulation of fibronectin and the downregulation of SMAD7 and TWIST1. We speculate whether the upregulation of wt-CFTR expression previously reported may be a way of compensating the emerging EMT that KLF4 KO causes, possible through a feedback mechanism to retain epithelial function.

Some of the impacts of KLF4 on differentiation and EMT have been associated with TGFβ signaling [57], which in turn can be linked to CFTR through NEDD4L [58] (Figure S4). In fact, CFTR has been previously linked to the TGFβ pathway, as TGFβ inhibits CFTR expression and individuals with CF have increased TGFβ signaling [59]. Therefore, we also characterize here the impact of KLF4 KO on the expression levels of some TGFβ signaling associated proteins (Figure 5). Overall, KLF4 KO seems to promote the expression of TGFβRI and RII, although this effect is only statistically significant in wt-CFTR cells. Analyses of SMADs levels show that phosphorylated (active) SMAD2 is increased in wt-CFTR cells and decreased in F508del-CFTR cells, while the TGFβ-inhibitory SMAD7 is decreased in F508del-CFTR cells. Altogether, these seem to indicate that KLF4 KO promotes TGFβ signaling in wt-CFTR cells at both the receptor and pSMAD2 levels, which may promote EMT. On the other hand, KLF4 KO has somewhat contrasting impacts on F508del-CFTR cells as it downregulates the expression of opposing factors (pSmad2 and Smad7), with a more unpredictable result in which noncanonical TGFβ signaling may also have an impact [60].

**Figure 8:**
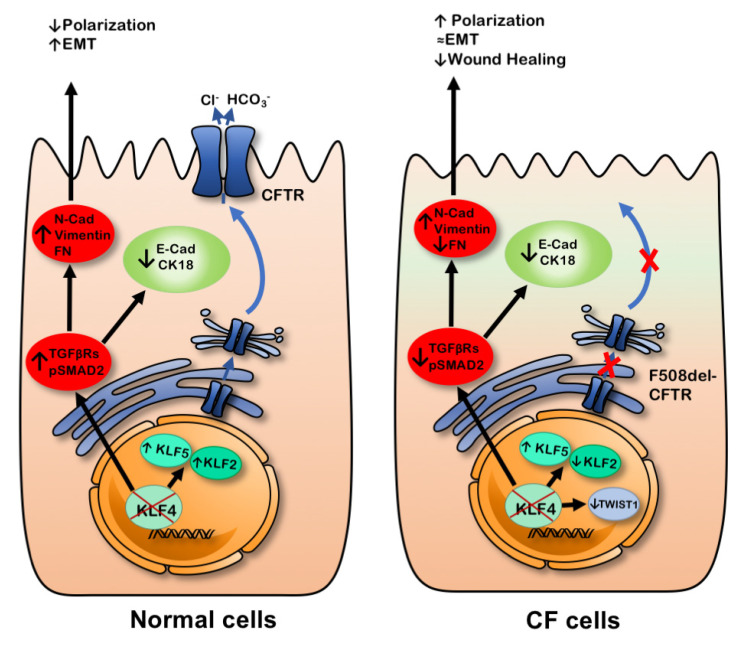
KLF4 KO seems to modulate the expression of several differentiation markers and promote a somewhat more mesenchymal phenotype. Nevertheless, there are markers that are differentially regulated in wt- vs. F508del-CFTR cells. Ultimately, KLF4 KO seemed to modulate polarization and wound healing depending on the CFTR status.

We then evaluated the levels of TWIST1, a transcription factor associated with EMT. In fact, TWIST1 was upregulated in CF cells, but the impact of KLF4 KO on TWIST1 levels is different in CF vs. non-CF cells as it decreases in F508del- but not in wt-CFTR cells. Given its role as a regulator of EMT [61], we speculate that this differential impact on TWIST1 levels may at least partially explain the different responses of wt- and F508del-CFTR CFBE cells to KLF4 KO. Figure 8 summarizes the different effects of KLF4 KO in wt- and F508del-CFTR cells.

Using a model for airway epithelial differentiation (BCi cells; Figure 6 and Figure 7), we observed that KLF2 expression decreases with BCi differentiation, being replaced by KLF4 and KLF5 expression, which peaks at 15 days of differentiation. We observed that GSK3β, EGFR, and pAKT display similar patterns of expression.

Altogether, these results shed some light on the impact of KLF4 in the cellular processes of differentiation, proliferation, wound healing, and EMT that are important in the context of CF. In our model, KLF4 does not have a major impact on proliferation by itself, but it should be regarded as a positive regulator of wound healing in F508del-CFTR cells, with a minor impact on wt-CFTR. Moreover, KLF4 seems to have different roles depending on CFTR status, as it appears to act as a positive regulator of polarization in wt-CFTR whilst acting as a negative regulator of polarization in F508del-CFTR cells. Despite having several effects on the levels of epithelial and mesenchymal markers and EMT-associated TFs, KLF4 seems to act as a negative regulator of EMT in wt-CFTR cells while having a less clear-cut impact on CF cells. Further studies, however, are required to fully address the role that KLF4 (or its downstream effectors) plays in those processes in order to propose it as a potential therapeutic target in the context of CF. This may be of particular interest in the correction of the developmental and differentiation/regeneration aspects that are disrupted in CF.

## 4. Materials and Methods

### 4.1. Chemicals, Antibodies, and Primers

Lists of primary and secondary antibodies used in WB are in Tables S1 and S2, respectively. Sequences for the primers used in qRT-PCR are in Table S3.

### 4.2. Cell Lines

CF-relevant immortalized bronchial epithelial cell lines, CFBE41o- (cystic fibrosis bronchial epithelial) cells, stably overexpressing wt- and F508del-CFTR, were used in this work. CFBE cells were grown in Eagle’s minimum essential medium (EMEM) with Earl salts and L-glutamine (Corning, 10-010-CVR, Corning, NY, USA), supplemented with 10% (*v*/*v*) fetal bovine serum (FBS; Gibco, 10270, Waltham, MA, USA), 1% pen/strep, and puromycin (Sigma-Aldrich, P8833, Taufkirchen, Germany) at 2.5 μg/mL for selection. To achieve polarization, cells were seeded on collagen IV precoated transwell permeable supports at a density of 1.25, 2.5, or 10 × 10^5^ cells, depending on the diameter of the filter (6.5, 12, or 24 mm insert, Corning 3470, 3460 and 3450, respectively, Corning, NY, USA). On the following day, media was changed from 10% to 2% (*v*/*v*) FBS to promote differentiation/polarization. Transepithelial electrical resistance (TEER) was measured regularly using a Millicell© ERS meter to evaluate polarization. The KLF4 knockout CFBE cell lines generated by the CRISPR–Cas9 technique were grown under the same conditions as the other CFBE cells.

BCi-NS1.1 cells were cultured with Pneumacult-Ex medium supplemented with Pneumacult-Ex 50X supplement (#05008; STEMCELL Technologies, Vancouver, BC, Canada), 96 μg/mL hydrocortisone (H0888; Sigma-Aldrich), and 1% penicillin–streptomycin (10,000 U/mL; 15140-148; Gibco) in a 37 °C, 5% CO_2_ humidified incubator. Following expansion, the cells were seeded onto either 6.5- or 12-mm diameter-size transwell inserts with 0.4 μm pore polyester membrane (#3470, #3460; Corning Incorporated), coated with human type IV collagen (C7521; Sigma-Aldrich) at a density of 1.5 × 105 or 3.0 × 105, respectively. Cells were cultured with 1:1 DMEM/F-12 (15-090-CM; Corning Incorporated) supplemented with 5% FBS (Gibco), 1% penicillin–streptomycin, 0.5% amphotericin B (15290-026; Gibco), and 0.1% gentamicin (G1272; Gibco). On the following day, the medium in both chambers was replaced with DMEM/F12 supplemented with 2% Ultroser G (15950-017; Pall Life Sciences, Port Washington, NY, USA), 1% penicillin–streptomycin, 0.5% amphotericin B, and 0.1% gentamicin. Air–liquid interface (ALI) was established once cells reached full confluency by aspirating the medium from the apical chamber. The medium was replaced every 2–3 d for 30 d, and polarization was monitored by measurements of TEER using a chopstick electrode (Millicell-ERS, Millipore, Burlington, MA, USA).

All cell lines were grown at 37 °C in 5% CO_2_.

### 4.3. TEER Measurements

TEER measurements were carried out on polarized CFBE and differentiated BCi-NS1.1 and pHBE cells, using a volt-ohmmeter (Millicell-ERS, Millipore, MER5000001) as a first indicator that the cells were differentiated and ready for further experiments. The volt-ohmmeter was allowed to incubate with the medium for 30 min at RT before measuring the resistance. The measurement was always performed before changing the media of the cells.

### 4.4. KLF4 KO Generation

Wt- and F508del-CFTR KLF4 KO cell lines were generated as previously described and validated by IF/WB and genomic sequencing, as previously described [20]. The validation of KO is shown in Figure S5.

### 4.5. Western Blot (WB)

For protein extraction, cells were washed three times with 1x PBS and lysed in 1x sample buffer (SB; 2x SB —Tris-HCl (Sigma, 30721, Taufkirchen, Germany) 62.5 mM, pH 6.8, SDS 3% (Gibco, 15553), glycerol 20% (Sigma, 92025), Bromophenol Blue 0.02% (*w*/*v*), DTT (Sigma, D0632; 100 mM) supplemented with protease inhibitor cocktail (Roche, 11697498001, Mannheim, Germany), 25 U benzonase (Sigma-Aldrich, #E1014-25G), and 3.125 mM of MgCl_2_ (Merck, 105833, Darmstadt, Germany). Lysates were prepared by repeated pipetting and then collected. The Bio-rad protein assay (Bio-Rad, 5000006, Hercules, CA, USA) was used to quantify protein extracts. This method is based on the Bradford method. Basically, the reagent is diluted in water (20:80) and a standard curve is made using different concentrations of BSA (Bio-Rad, 5000002), applying 0–20 µL of the standard into 1000–980 µL of the reagent. Then, 10 µL of the samples is applied to 990 µL of the reagent. All of these are incubated for 5 min at RT and assessed in a spectrophotometer by measuring the absorbance at 595 nm. Standards are used to create a standard linear curve, and the concentration of the samples is calculated by plotting the results against the standard curve.

Then, 25–40 μg of protein is loaded onto polyacrylamide gels (4% for stacking and 7% or 10% for resolving gels) in order to perform SDS/PAGE. Transfer onto polyvinylidene difluoride (PVDF) membranes (Merck Millipore, IPVH00010, Burlington, MA, USA) was performed using a wet-transfer system. The membranes were blocked for 1 h with 5% (*w/v*) non-fat milk (NFM) in PBS supplemented with Tween 20 (Fisher BioReagents, BP337-100, Hampton, NH, USA). This was followed by incubation with the primary antibody overnight at 4 °C, with gentle shaking. Horseradish peroxidase (HRP)-conjugated secondary antibodies were applied for 1 h at RT. All the antibodies were diluted in the blocking solution. Membrane luminescence was detected on a Chemidoc XRS+ system (Bio-Rad, 170-8265). Quantification of band intensity was performed using Image Lab software (Bio-Rad, 170-9690), which integrates peak area.

All measurements were normalized against loading controls. A list of primary and secondary antibodies can be found in Tables S1 and S2.

### 4.6. RT-qPCR

Total RNA was extracted using the NZY total RNA isolation kit (NZYtech MB13402, NZYtech, Lisbon, Portugal) according to the protocol provided. Then, a mix containing forward and reverse primers, cDNA (5 ng), and Evagreen SsoFast PCR reagent (Bio-Rad, 172-5204) was used along with a Bio-Rad CFX96 system. Bio-Rad CFX Manager 2.0 software (Bio-Rad, 1845000) was used for analysis. A standard cycle protocol was used for PCR amplification (1 min at 95 °C, followed by 40 cycles of 10 s at 95 °C and 30 s at 60 °C). Technical duplicates were used in amplification, melt curves were examined to confirm the amplification of specific products, and negative controls were confirmed to be free of amplification after 40 PCR cycles. Mean relative levels of expression were calculated for the target genes using the ∆∆CT method, where fold change = 2^−∆∆CT^, using mean levels of expression in non-CF samples as the baseline (or wt-CFTR cells). Information regarding the primer sequences used is presented in Table S3. ACTB and GAPDH were used as housekeeping genes for the experiments on lung and cells (BCi and CFBE), respectively.

### 4.7. Growth Curves

For CFBE cells, 50,000 CFBE cells were seeded in a P24 well plate. After 24 h, pictures were then taken at given timepoints using the brightfield microscope (Leica Microsystems, Wetzlar, Germany) at given timepoints, and the cell number was counted in the central area using ImageJ for image analysis.

### 4.8. Immunostaining

Proliferation was assessed by the evaluation of Ki-67 positive staining. Briefly, 100,000 cells were seeded in P24 well plates and grown until fully confluent (circa 24 h). A wound was performed by scratching with a pipette tip, and cells were grown for 24 h. Then, cells were fixed with PFA for 20 min and left overnight with PBS at 4 °C. Cells were then permeabilized with 0.3% Triton X-100 solution for 15 min and treated with ammonium chloride 0.5 M for 15 min to reduce background. Cells were then blocked with PBS 2% BSA for 30 min, followed by 90 min of incubation with primary antibody against Ki-67 (1/100—M7240 Dako™). Cells were then washed with PBS and incubated with DAPI (1/20—Amersham PA53021) and a secondary antibody for 1 h (1/1500—Alexa568^®^ anti-mouse). After washing with PBS, images were taken at a fluorescence microscope, and the Ki-67 positive ratio was assessed.

For SiR-Actin staining of the cells at the wound edge, cells were seeded on 96-well plates and grown until confluent. Cells were then wounded using a 96-pin tool, and wound closure was allowed for 16 h before cells were fixed and stained using SiR-Actin (Spirochrome, Switzerland) and Hoechst. Cells were imaged at 20× using a Leica SPE microscope (Leica, Jena, Germany).

### 4.9. Wound Healing

Fully polarized CFBE cells were mechanically injured by scraping a sterile P10 pipette tip across the cell monolayer. For cell wounding, PBS was added to the apical side of the filters. After wounding, the apical surface was washed twice with PBS to remove cell debris. Fresh media was added to the basolateral and apical sides.

Wound closure was monitored by live-cell imaging (24 h, 37 °C, 5% CO_2_) with an automated Leica SP8 confocal microscope. Images were taken every hour. The software used for the acquisition was Leica’s LAS x (Leica, Jena, Germany), and image processing was performed on ImageJ FIJI. FIJI was used to segment and measure the wound area. Wound closure was then calculated as $Wound\;size\left(\%\right)=\left(A_{t}/A_{0}\right)\times 100$, where $A_{t}$ is the area for a given time point and $A_{0}$ is the initial wound area. Wound size was plotted as a function of time (h) and was used to calculate the rate (slope) of wound closure (%/h).

### 4.10. Statistical Analyses

Data are presented as mean ± SEM. Student’s *t*-test for unpaired samples was used for statistical analyses. Prism 6 software (GraphPad, Inc., San Diego, CA, USA) was used for graph design and statistical analyses. Significant differences were defined for *p* ≤ 0.05 and marked with an asterisk. Other trends or tests may be stated in the legend. *n* = 3 unless stated otherwise in the figures or legends. Comparisons are only made between WB from the same blot.

## Supplementary Materials

The following are available online at https://www.mdpi.com/1422-0067/21/18/6717/s1, Figure S1: KLF4 KO does not have major impact on the expression levels of Ki-67. Figure S2: Actin staining of cells at the wound front in wt- and F508del-CFTR CFBE cells and their respective KLF4 KO counterparts. Figure S3: KLF4 KO showed no major impact on the levels of several differentiation markers and transcription factors. Figure S4: Signalling pathways connecting TGFβ to CFTR as displayed in STRING database. Figure S5 KLF4 KO validation. Table S1: Primary antibodies information. Table S2: Secondary antibodies information. Table S3: Primers information.

## Abbreviations

CF	Cystic fibrosis
CFBE	Cystic fibrosis bronchial epithelial
CFTR	Cystic fibrosis transmembrane conductance regulator
CK	Cytokeratin
Clnx	Calnexin
Cx	Connexin
DSPI/II	Desmoplakin I/II
E-Cad	Epithelial cadherin
EGFR	Epidermal growth factor receptor
EMT	Epithelial to mesenchymal transition
F508del	Deletion of phenylalanine at position 508
GAPDH	Glyceraldehyde 3-phosphate dehydrogenase
GSK3β	Glycogen synthase kinase 3 beta
KLF	Kruppel-like factors
KO	Knock out
PM	Plasma membrane
TEER	Transepithelial electrical resistance
TF	Transcription factor
TGF	Transforming growth factor
WB	Western blot
wt	Wild-type
ZO-1	Zonula occludens-1
