# Supplementary material for: Impact of KLF4 on Cell Proliferation and Epithelial Differentiation in the Context of Cystic Fibrosis
Source: Int J Mol Sci. 2020 Sep 14;21(18):6717. doi: 10.3390/ijms21186717 (PMC7555189; doi:10.3390/ijms21186717)
Supplement: Supplementary file 1 [file ijms-21-06717-s001.zip › Table supplementary materials.pdf]

Table S1 – Primary antibodies information

Target	Application	Dilution	Host	MW (kDa)	Company	Reference
KLF2	WB	1/200	Mouse	37	Abcam	ab194486
KLF4	WB	1/1000	Rabbit	60	Santa Cruz	sc-20691
KLF5	WB	1/500	Rabbit	50	Abcam	ab24331
GAPDH	WB	1/10000	Mouse	37	Abcam	ab8245
E-Cadherin	WB	1/5000	Mouse	135	BD Biosciences	610181
N-Cadherin	WB	1/1000	Mouse	135	BD Biosciences	610920
Calnexin	WB	1/3000	Mouse	90	BD Biosciences	610523
Vimentin	WB	1/1000	Rabbit	60	Abcam	ab92547
Fibronectin 1	WB	1/1000	Mouse	250	Santa Cruz	sc-8422
Cytokeratin 18	WB	1/100	Mouse	45	Santa Cruz	sc-323229
ZO1	WB	1/5000	Mouse	250	Invitrogen	33-9100
TGFbetaRI	WB	1/200	Rabbit	50	Cell signaling	3712s
TGFbetaRII	WB	1/200	Mouse	80	Santa Cruz	sc-17719
pSmad2	WB	1/200	Rabbit	60	Cell signaling	3101s
Smad7	WB	1/1000	Rabbit	50	Aviva	ARP32008
TWIST1	WB	1/50	Mouse	25	Abcam	ab50887
Vinculin	WB	1/1000	Mouse	130	Santa Cruz	sc-73614
Ki67	WB	1/2500	Rabbit	250	Abcam	ab16667
	IF (Ki67+)	1/100	Mouse		Dako	M7240
Cytokeratin 5	WB	1/1000	Rabbit	60	Invitrogen	PA1-37974
Cytokeratin 13	WB	1/500	Goat	50	Santa Cruz	sc-31703
Cytokeratin 14	WB	1/500	Mouse	50	Santa Cruz	sc-53253
TEAD4	WB	1/200	Mouse	50	Abcam	ab58310
Claudin1	WB	1/100	Mouse	20	Santa Cruz	sc-166338
DSPI/II	WB	1/500	Rabbit	250	Santa Cruz	sc-33555
Connexin 31	WB	1/200	Mouse	35	Santa Cruz	sc-81803
SNAIL+SLUG	WB	1/1000	Rabbit	25	Abcam	ab180714
Occludin	WB	1/100	Mouse	90	Santa Cruz	sc-133256
pAKT	WB	1/1000	Rabbit	60	Cell signaling	13038s
Connexin 30	WB	1/200	Mouse	30	Santa Cruz	sc-514847

Table S2 – Secondary Antibodies information

Antibody	Target	Use	Dilution	Host	Company	Reference
(H+L)-HRP Conjugate	Mouse IgG	WB	1/3000	Goat	Bio-Rad	170-6515
(H+L)-HRP Conjugate	Rabbit IgG	WB	1/3000			170-6516
(H+L)-HRP Conjugate	Goat IgG	WB	1/5000	Donkey	Invitrogen	A15999

Table S3 – Primers Information

Target	Fw/Rv primer	Sequence (5'-3')
ACTB	Forward	CTCTTCCAGCCTTCCTTCCT
	Reverse	AGCACTGTGTTGGCGTACAG
KLF2	Forward	CTACACCAAGAGTTCGCATCTG
	Reverse	CCGTGTGCTTTCGGTAGTG
KLF4	Forward	CCCACATGAAGCGACTTCCC
	Reverse	CAGGTCCAGGAGATCGTTGAA
KLF5	Forward	GAACTGGTCTACGACTGAGGC
	Reverse	CCTGGTCCAGACAAGATGTGA
GAPDH	Forward	ATGGGGAAGGTGAAGGTCG
	Reverse	GGGGTCATTGATGGCAACAATA
