# Supplementary figures and images for: Impact of KLF4 on Cell Proliferation and Epithelial Differentiation in the Context of Cystic Fibrosis
Source: Int J Mol Sci. 2020 Sep 14;21(18):6717. doi: 10.3390/ijms21186717 (PMC7555189; doi:10.3390/ijms21186717)

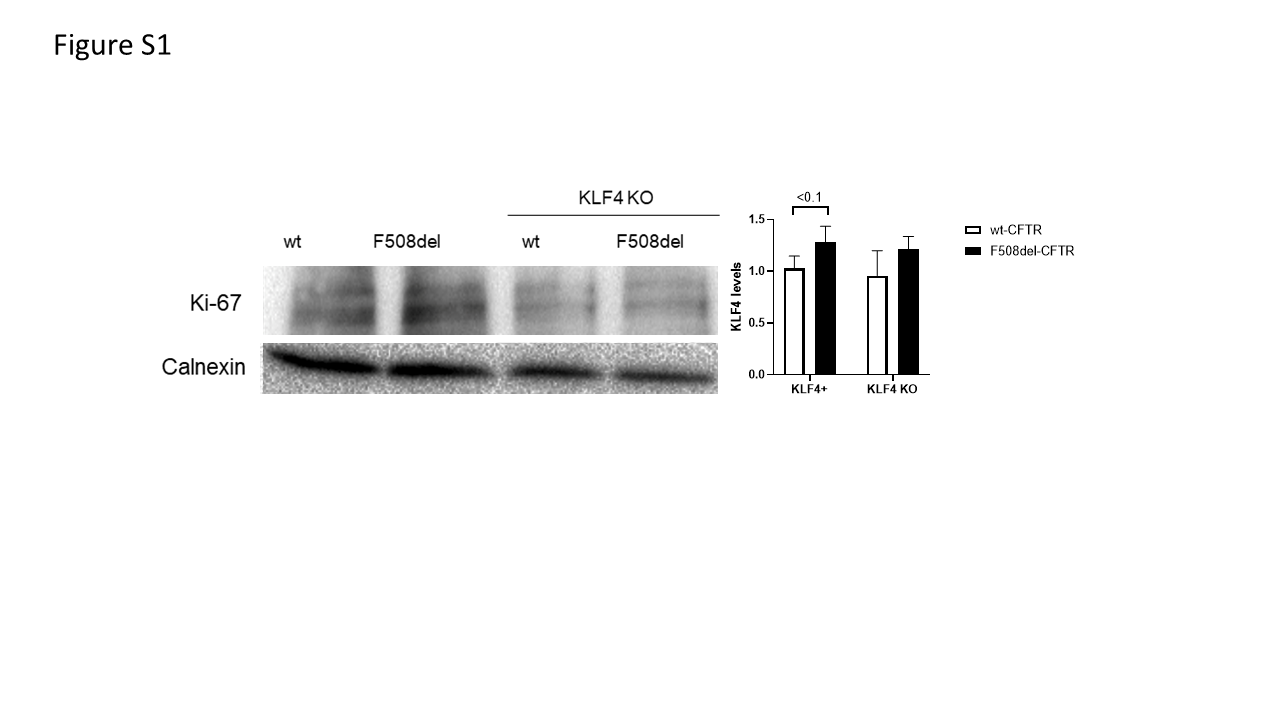

Supplement: Supplementary file 1 [file ijms-21-06717-s001.zip › FigS1_reupload.tif]

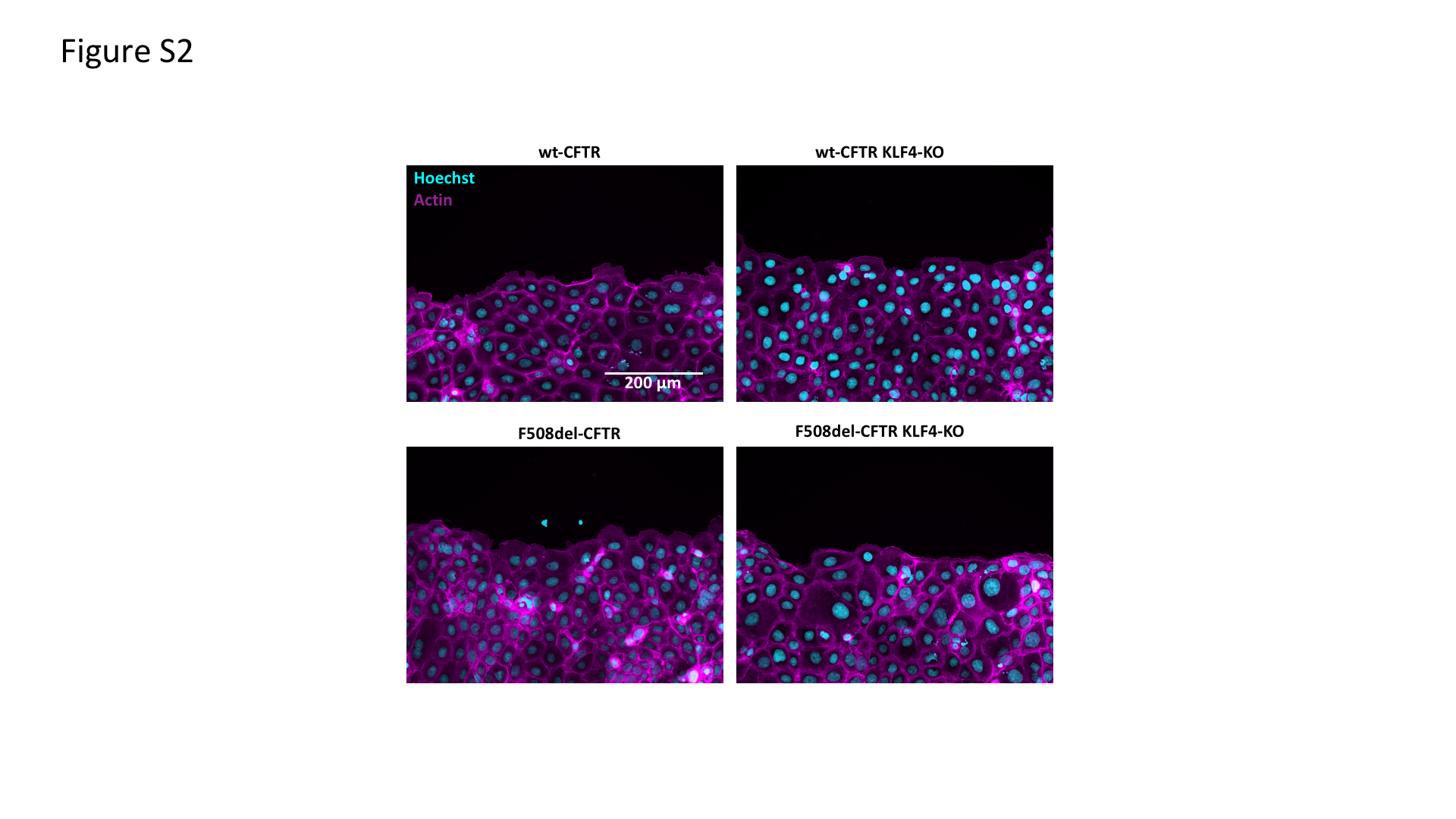

Supplement: Supplementary file 1 [file ijms-21-06717-s001.zip › FigS2.tiff]

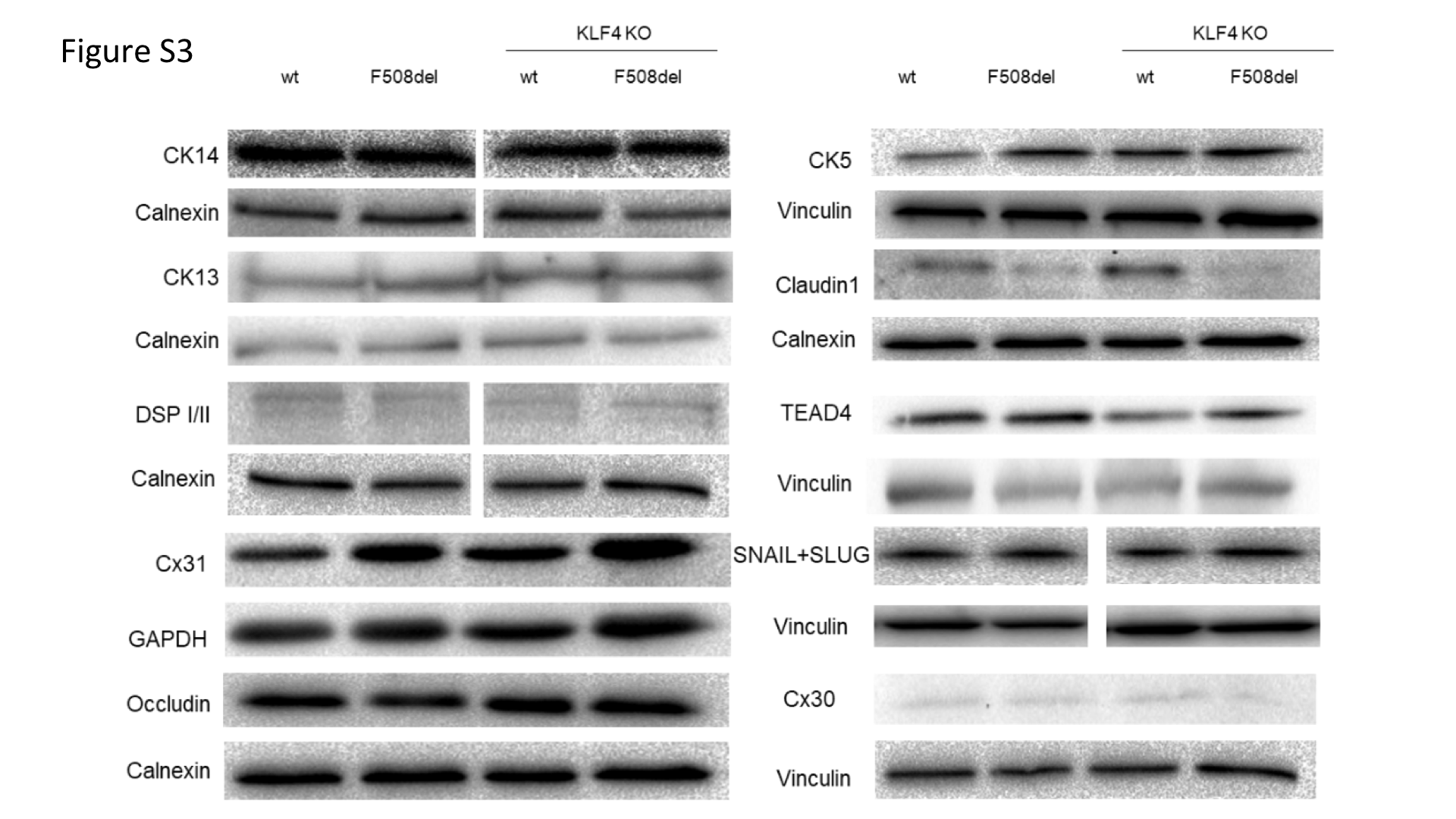

Supplement: Supplementary file 1 [file ijms-21-06717-s001.zip › FigS3.tiff]

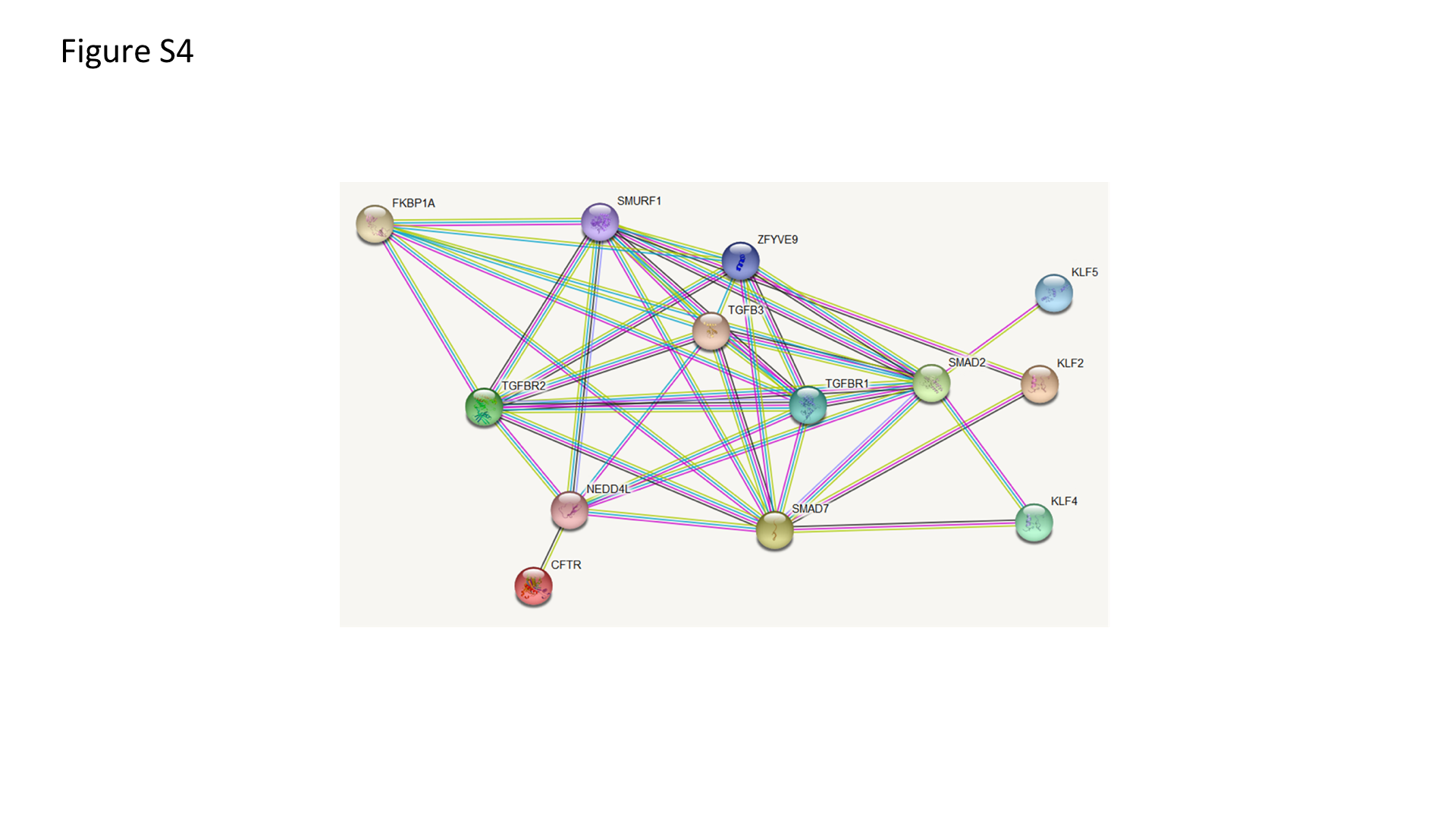

Supplement: Supplementary file 1 [file ijms-21-06717-s001.zip › FigS4.tiff]

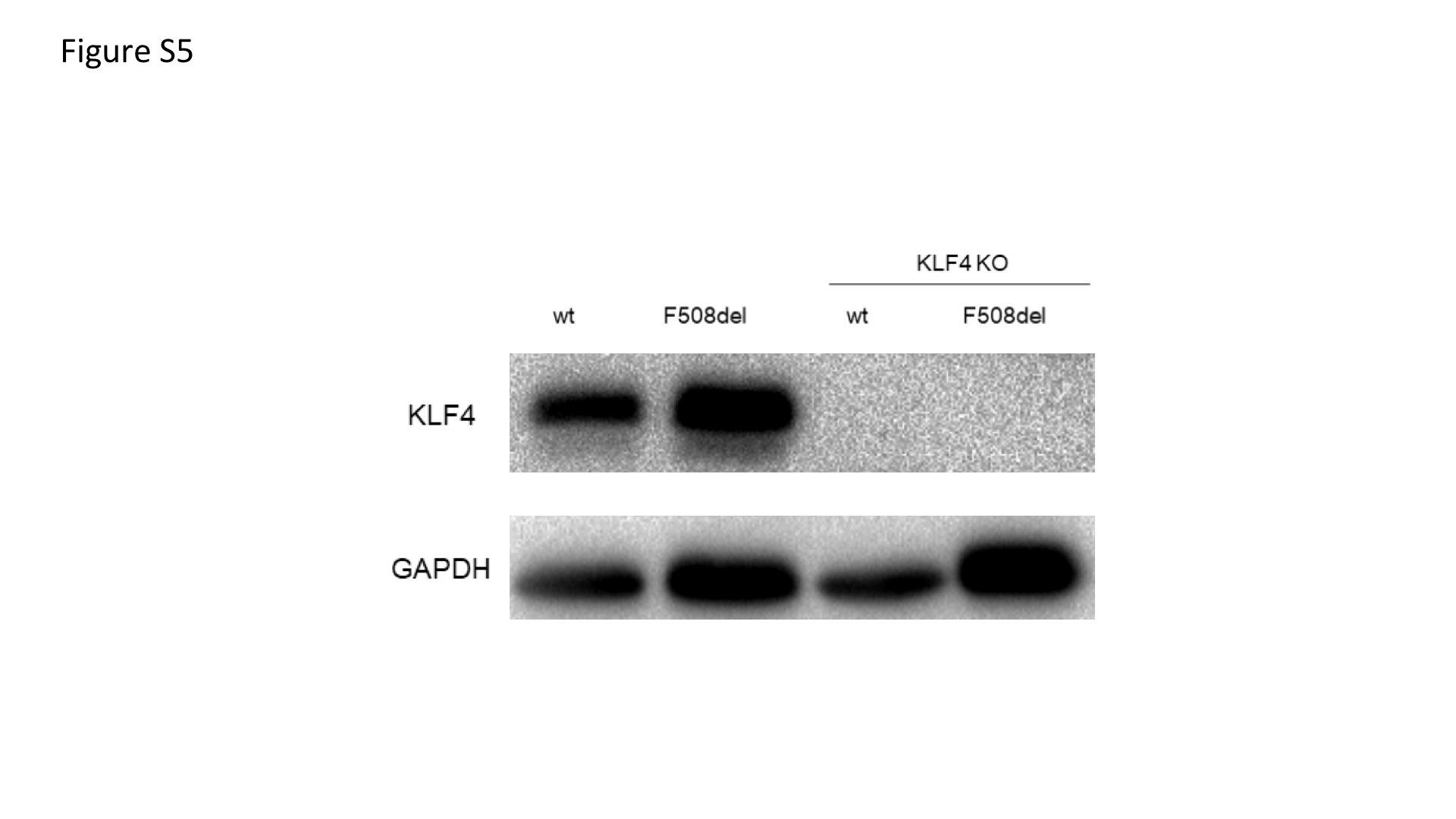

Supplement: Supplementary file 1 [file ijms-21-06717-s001.zip › FigS5.tiff]
